# Clinical evaluation of the efficacy of limbus artificial intelligence software to augment contouring for prostate and nodes radiotherapy

**DOI:** 10.1093/bjr/tqae077

**Published:** 2024-04-16

**Authors:** Alison Starke, Jacqueline Poxon, Kishen Patel, Paula Wells, Max Morris, Pandora Rudd, Karen Tipples, Niall MacDougall

**Affiliations:** Radiotherapy Physics, St Bartholomew’s Hospital, London EC1A 7BE, United Kingdom; Radiotherapy Physics, St Bartholomew’s Hospital, London EC1A 7BE, United Kingdom; Clinical Oncology, St Bartholomew’s Hospital, London EC1A 7BE, United Kingdom; Clinical Oncology, St Bartholomew’s Hospital, London EC1A 7BE, United Kingdom; Radiotherapy Physics, St Bartholomew’s Hospital, London EC1A 7BE, United Kingdom; Clinical Oncology, St Bartholomew’s Hospital, London EC1A 7BE, United Kingdom; Clinical Oncology, St Bartholomew’s Hospital, London EC1A 7BE, United Kingdom; Radiotherapy Physics, St Bartholomew’s Hospital, London EC1A 7BE, United Kingdom; Barts Cancer Institute, Queen Mary University of London, London EC1M 6BQ, United Kingdom

**Keywords:** AI, contour, radiotherapy, prostate, machine learning, planning, oncology

## Abstract

**Objectives:**

To determine if Limbus, an artificial intelligence (AI) auto-contouring software, can offer meaningful time savings for prostate radiotherapy treatment planning.

**Methods:**

Three clinical oncologists recorded the time taken to contour prostate and seminal vesicles, lymph nodes, bladder, rectum, bowel, and femoral heads on CT scans for 30 prostate patients (15 prostate, 15 prostate and nodes). Limbus 1.6.0 was used to generate these contours on the 30 CT scans. The time taken by the oncologists to modify individual Limbus contours was noted and compared with manual contouring times. The geometric similarity of Limbus and expert contours was assessed using the Dice Similarity Coefficient (DSC), and the dosimetric impact of using un-edited Limbus organs at risk contours was studied.

**Results:**

Limbus reduced the time to produce clinically acceptable contours by 26 minutes for prostate and nodes patients and by 13 minutes for the prostate only patients. DSC values of greater than 0.7 were calculated for all contours, demonstrating good initial agreement. A dosimetric study showed that 5 of the 20 plans optimized using unmodified AI structures required unnecessary compromise of PTV coverage, highlighting the importance of expert review.

**Conclusions:**

Limbus offers significant time saving and has become an essential part of our clinical practice.

**Advances in knowledge:**

This article is the first to include bowel and lymph nodes when assessing potential time savings using Limbus software. It demonstrates that Limbus can be used as an aid for prostate and node radiotherapy treatment planning.

## Introduction

Radiotherapy treatments are individualized to a patient’s anatomy and disease, with each of these being visualized by CT (and/or) MRI imaging. A treatment planning system (TPS) is used to simulate the transport of radiation in the patient’s body, the aim being to focus the highest doses of radiation on the patient’s cancer and minimize the radiation dose to organs near the cancer. However, expert user intervention is required to outline these volumes. This is a time-consuming process which is a common bottleneck in the treatment planning pathway and as with all manual processes is susceptible to inter-observer variation.^1^^,^^2^

The use of artificial intelligence (AI) models trained to contour these structures is a rapidly expanding field aiming to automate this process. If sufficiently accurate and safely implemented, these AI models have the potential to deliver meaningful efficiencies to the radiotherapy planning process. As a secondary benefit they could help in the effort to standardize contours.

Recent studies have shown promising results validating AI based models for a range of treatment sites.^3–7^ Many different evaluation methods have been employed to judge AI auto-contouring software accuracy,^8^ with geometric similarity of expert and auto-generated contours being one of the commonly used comparisons^9–11^. These geometric indices provide a crude measure of the efficacy of AI contouring software,^5^ but despite their known issues we included them to provide context to existing studies.

However, perfect generation of AI structures is unlikely, due to the wide tails of the population distribution of human anatomical variations, meaning expert input continues to be required to review auto-contours for safety reasons: so called “human in the loop”. As such, our focus is investigating the potential time saving offered to an expert working alongside an AI model in routine clinical practice. Some studies have quantified this time saving, with reductions ranging from 30% to 56%^4^^,^^12–14^.

These potential efficiency gains led to our clinic deciding to validate a commercially available solution: Limbus. The Limbus software (Limbus AI Inc, Regina, SK, Canada) was selected as it is a locally installed program that does not require an internet connection. This has many benefits such as no patient data is shared with a third party, making information governance compliance simpler; the program version is under local control, so there are no version control or program creep issues and it runs on local hardware without the need for expensive graphics cards. Initial testing was performed on prostate patients as this cohort of patients represents a significant proportion of those treated meaning any time saving offered would have an immediate impact (18% of radiotherapy patients in England between April 2021 and March 2022 were prostate patients^15^).

Many studies comparing expert and AI contours for prostate patients have been conducted for a range of algorithms^5^^,^^12–14^^,^^16^^,^^17^. Of those validating Limbus software, all compared the geometric similarity of clinician and AI structures^11^^,^^13^^,^^18^^,^^19^; a number looked at potential time savings^13^^,^^19^ but none included pelvic lymph nodes or bowel. These structures typically take the longest to contour so have the largest potential time savings.

The primary end point of this study was to determine if Limbus AI software can offer meaningful time savings to the prostate planning pathway for patients both with and without nodal volumes, and be used in a way that does not raise clinical risk levels. We also studied clinician satisfaction with the contours, potential dosimetric risks of using un-modified AI contours, and assessed the geometric accuracy of the auto-generated contours to enable comparison with other auto-contouring solutions.

## Methods

### Study design

Anatomical structures were contoured manually on a blank CT dataset, by Consultant Clinical Oncologists, and peer reviewed. Limbus contours were then applied to the patient CT sets. The Limbus contour scoring was then completed independently by two different FRCR part 2 qualified Oncologists on a dataset with Limbus contours only. Modifications to limbus contours were completed by the consultant oncologists after a 3 month gap to reduce memory bias of contouring, on a dataset with Limbus contours only.

### Patient selection

The study used prospective patient data, gathering data from the start of the project onwards, with 30 patient CT scans (15 prostate, and 15 prostate and nodes) acquired using a Siemens Somatom Confidence 64 scanner (Siemens Healthineers, Erlangen, Germany) using the following parameters: 120 kV, 2 mm slice thickness and an in-plane pixel size of 1 mm × 1 mm. Patients were scanned with a full bladder and an empty rectum, as per department protocol, with CT data sets sent directly to Aria v17.0 (Varian Medical Systems, Palo Alto, CA) for clinician contouring. The clinical target volume (CTV) was drawn as prostate and seminal vesicles, with organs at risk (OARs) bladder, rectum and femoral heads outlined following the PACE clinical trial protocol.^20^ In addition, for the nodes patients, bowel and pelvic lymph nodal volumes were also contoured, with lymph nodes outlined following guidelines published by Taylor et al^21^.

### Auto-contouring

Auto-contours were generated on each of the CT data sets using Limbus v1.6.0 (Limbus AI Inc, Regina, SK, Canada): an AI-coded, python-based software. The Limbus model uses a convolution neural network algorithm which takes input images and assigns importance to different features; this enables it to differentiate structures within an image. Additionally, its U-net model architecture enables it to be trained with fewer images. The Limbus contouring software is trained using TensorFlow on publicly available CT data sets^11^. For the purpose of this study, Limbus was set to produce outlines for prostate and seminal vesicles, lymph nodes (if appropriate), bladder, rectum, bowel, and femoral heads. Contours for these patients were generated in less than 2 minutes and the structure sets were exported to Eclipse for review.

### Clinician scoring of Limbus contours

Two FRCR part 2 qualified clinical oncologists visually assessed all the Limbus contours for the patient scans and graded their quality on a 0-3 scale^22^. A grading of 3 meant no editing of the contour was required. Quality scores of 2 and 1 were defined as <4 slices and ≥4 slices required editing respectively, while unacceptable contours were graded 0. Median scores for each OAR were reported to gain a basic understanding of clinician scoring.

### Geometric similarity

The geometric similarity of the expert and unmodified Limbus contours was assessed using the Dice Similarity Co-efficient (DSC) and 95% percentile Hausdorff Distance (HD). DSC describes the overlap of two structures with values ranging from 0 to 1, with 1 denoting a perfect overlap of structures, and 0 no overlap at all. The HD is defined as the maximum distance between two structures. This measure is very sensitive to outliers, therefore the 95% HD was used, which is the 95^th^ percentile of all distances between the two contours. Contour analysis (v0.0.14) software was used (Limbus). Median DSC and 95% HD values were reported, along with inter-quartile ranges (IQR) for each structure. A DICE of >0.7 was chosen to indicate adequate similarity.

### Timing study

The median time taken to outline individual organs *de novo* was compared with the median time taken to review, and modify, the automatically generated contours. Three FRCR part 2 qualified clinical oncologists timed themselves contouring individual OARs and CTVs on the blank CT datasets. Time to outline individual organs was measured using an in-house coded excel spreadsheet, writing to an Access database back end. The software allowed the clinician to time themselves drawing individual structures, as well as pause the timer if interrupted whilst contouring. The same three oncologists then timed themselves modifying the Limbus structures on the CT sets 3 months later.

### Dosimetric impact of using automatically generated contours

To investigate the impact of using unmodified AI contours in a clinical setting, the dosimetric implications of using un-edited, auto-generated structures for treatment planning was assessed using the patient scans for clinician scoring and geometric analysis.

The 20 clinically approved VMAT treatment plans were re-optimized using the unmodified AI generated Limbus OARs and the oncologist approved PTVs. PTV coverage was compromised (aim to cover 98% of the PTV with 95% of the prescription dose) to ensure OAR dose maximum limits complied with departmental protocols where necessary. This plan was then re-calculated on the clinically edited structure set and the doses reported. Doses were compared for Limbus and clinical contours for bladder, rectum, femoral heads and bowel for each plan. PTV coverage was noted for each plan, and compared with that of the original clinical plan.

All plans were calculated using the Eclipse 6 MV AAA algorithm (version 17.0, Varian Medical Systems, Palo Alto, CA). The prostate and seminal vesicles were prescribed to receive 60 Gy in 20 fractions. Nodal volumes were planned to receive 47 Gy in 20 fractions.

## Results

### Clinician scoring of Limbus contours

The median scoring of the two clinicians for the Limbus contours is shown in Table 1. The scoring results show that the clinicians typically had to edit Limbus’ contours on four or more CT slices for the femoral heads, bowel and pelvic lymph nodes. Fewer slices required editing for the bladder, rectum and prostate. No systematic difference between the two clinicians’ scorings was noted.

**Table 1.** Clinician assessment of Limbus contours.

Bladder	Rectum	Fem head R	Fem head L	Bowel	Prostate and seminal vesicles	Pelvic lymph nodes
2	2	1	1	1	2	1

The clinicians reported differences between the Limbus contours and their own for each OAR and the lymph nodes; these are described below. No systematic differences were seen between the experts and Limbus for the prostate and seminal vesicle outlines.

### Bladder

**Figure 1.**
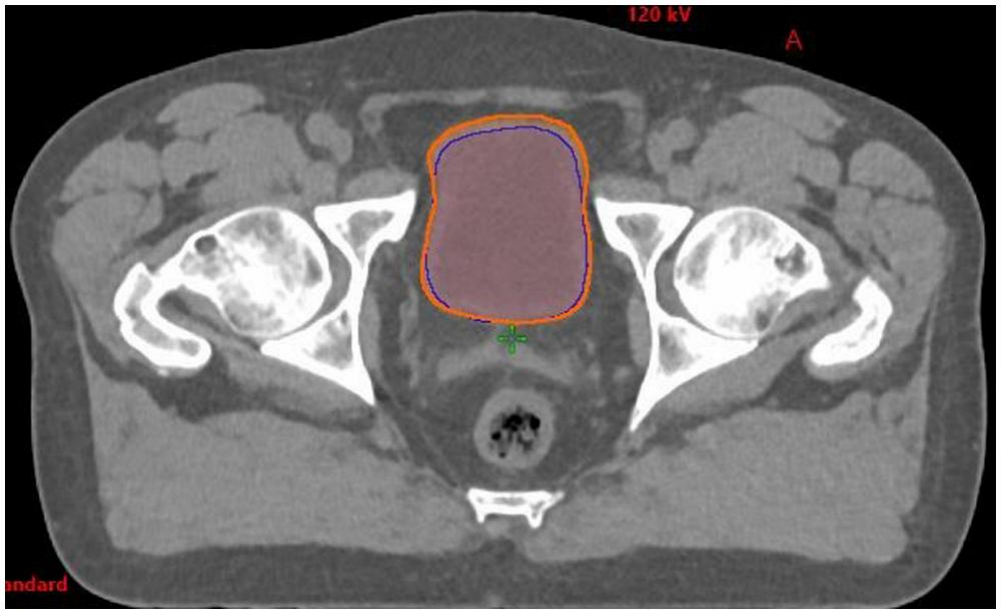
Transverse view of bladder contours: Limbus (dark blue), clinical oncologist (orange).

Clinicians were satisfied with Limbus contours when there were no organs adjacent to the bladder. It was noted that Limbus missed part of the bladder when another organ was superior or posterior to it (ie, bowel or rectum) (Figure 1).

### Rectum

**Figure 2.**
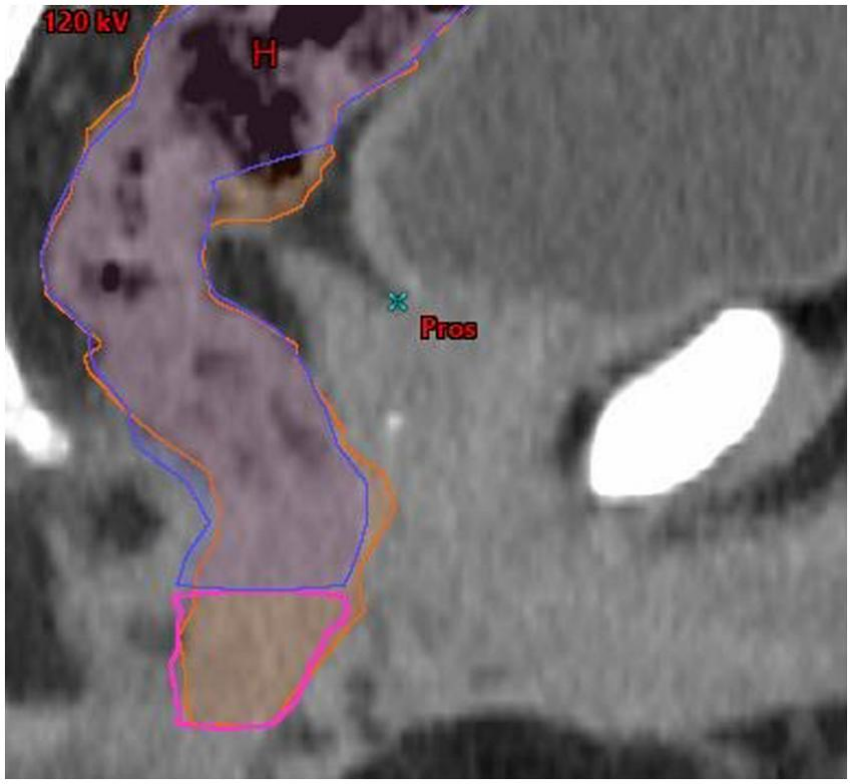
Sagittal view of rectum contours: Limbus rectum (dark blue), Limbus anal canal (magenta), clinical oncologist rectum (orange).

The modifications required to ensure Limbus’ rectal contours comply with the PACE protocol were relatively minor. PACE defines the entire anorectum as rectum. Clinicians therefore combined Limbus’ anal canal and rectum structures to generate a rectum structure. The inferior end of this was then cropped to the tip of the ischial tuberosity and the superior end was cropped to the level of the rectosigmoid junction (Figure 2).

### Femoral heads

**Figure 3.**
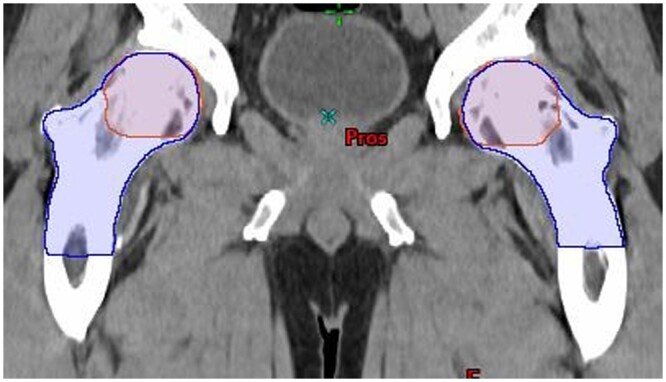
Coronal view of femur contours: Limbus (dark blue), clinical oncologist (orange).

Agreement between the clinicians and Limbus was good at the femoral head level. The main difference was Limbus contours both the femoral head and neck, whereas clinicians only contour the femoral head, as per PACE protocol (Figure 3). Typically, 5-6 cm of slices were deleted from the Limbus contours, explaining the score of 1.

### Bowel

**Figure 4.**
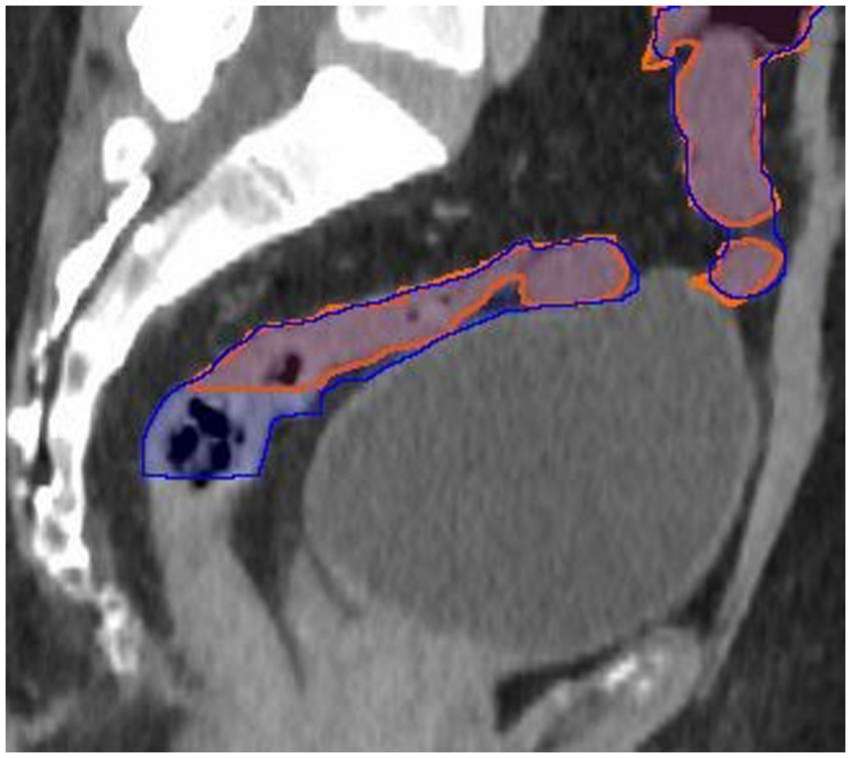
Sagittal view of bowel contours: Limbus (dark blue), clinical oncologist (orange).

Limbus overestimated the inferior extent of the bowel, with the structure typically needing four or more slices deleted inferiorly (Figure 4). Inaccuracies were also noted when bowel was adjacent to the bladder. Vessels and muscle were sometimes included in the Limbus bowel structure.

### Pelvic lymph nodes

Taylor et al^21^ demonstrated that a modified 7 mm margin around blood vessels offers a good surrogate for the pelvic lymph node CTV. Limbus lymph nodes were observed to be tighter than this, meaning clinicians had to expand this margin on the AI contours. The AI lymph nodes extended too far inferiorly into the lower pelvis, stopping inferior to the obturator fossa. It was also observed that the presacral bridge extends (correctly) to the superior border of S3. However, it stops contouring at this point. Individual presacral regions should be contoured to the bottom of S3.

### Geometric similarity

The DSC for all contours was greater than 0.7, meaning they showed relatively good agreement (Table 2). The median DSC for the bladder showed an almost perfect overlap of the Limbus and clinician drawn bladder contours. The median DSCs for rectum and bowel were 0.82 and 0.83, respectively. Similar results were seen for the two femoral heads, with median DSC values of 0.80 and 0.78 for the left and right respectively. The prostate DSC value of 0.85 again indicated good overlap of the Limbus and expert prostate. The level of agreement seen for the pelvis lymph nodes was poorer.

**Table 2.** Median values, interquartile range shown in brackets.

	DSC	95% HD (mm)
Bladder (*n* = 30)	0.96 (0.95-0.97)	2.8 (2.6-3.6)
Rectum (*n* = 30)	0.82 (0.79-0.85)	9.7 (7.9-10.8)
Left femoral head (*n* = 30)	0.80 (0.76-0.80)	20.8 (17.7-23.2)
Right femoral head (*n* = 30)	0.78 (0.75-0.81)	19.9 (18.9-23.6)
Bowel (*n* = 15)	0.83 (0.79-0.87)	12.0 (5.8-16.0)
Prostate and seminal vesicles (*n* = 20)	0.85 (0.82-0.88)	4.8 (4.0-5.5)
Pelvic lymph nodes (*n* = 15)	0.71 (0.69-0.72)	13.5 (12.0-22.3)

The 95% HD values are relatively large for all structures except the bladder. The combination of DSC scores greater than 0.7, and 95% HD values greater than or close to 10 mm for all but the bladder and prostate structures corresponds to the feedback from the clinicians: good agreement on slices where experts and Limbus both contoured, with the main difference seen in the superior-inferior levels.

### Contour timing: manual vs limbus

A clear reduction in contouring time was seen with the introduction of AI contours, shown in Table 3. The overall time required for prostate and node patients was reduced from approximately 56 to 29 minutes, representing a time saving of 47%. Similarly for prostate only patients the overall expert time needed decreased from 21 to 7 minutes. The biggest efficiency gains were seen for the bowel, pelvic lymph nodes and prostate, with reductions in contouring time of 7, 5 and 5 minutes, respectively. There was no systematic difference in outlining time between the three clinicians.

**Table 3.** Contouring times required to draw structures from scratch with time to modify Limbus structures.

	*de novo* contour time (min: s)	Edit time (min: s)	Time difference (min: s)
Bladder	4:00 (3:07-4:39)	0:50 (0:30-1:10)	3:10
Rectum	4:40 (4:02-5:21)	1:00 (0:59-1:41)	3:40
Femoral head LT	1:30 (1:07-2:04)	0:45 (0:40-0:58)	0:45
Femoral head RT	1:34 (1:14-2:06)	0:42 (0:40-1:02)	0:51
Bowel	14:10 (12:04-16:38)	6:48 (6:19-7:38)	7:23
Prostate and seminal vesicles	9:21 (6:00-12:32)	4:10 (1:59-5:00)	5:11
Pelvic lymph nodes	20:37 (20:00-21:45)	15:00 (13:16-16:52)	5:37
Total time for prostate only (excludes bowel and nodes)	21:05	7:27	13:38
Total time for prostate and nodes	55:52	29:15	26:37

Median values shown with inter-quartiles in brackets.

### Dosimetric impact of using automatically generated contours

**Figure 5.**
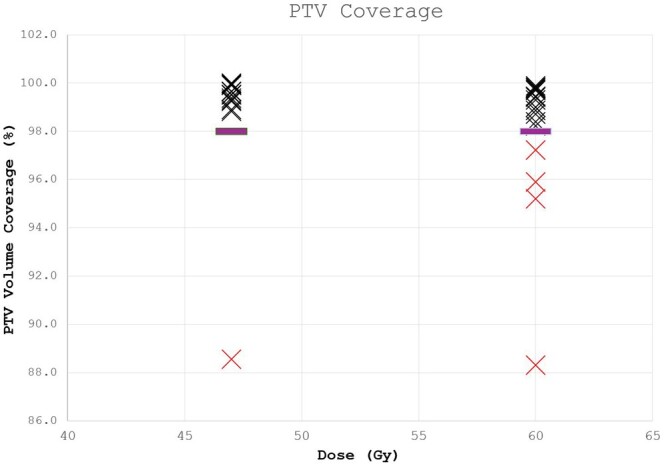
PTV coverage (PTV60Gy and PTV47Gy) for plans optimized using AI generated OARs. The purple line shows required coverage for each PTV (98%).

Figure 5 shows the dosimetric impact using unmodified AI OAR contours has on PTV coverage for prostate (± nodes) plans. 5 of the 20 plans (25%) re-optimized using Limbus OARs required PTV coverage to be compromised to achieve the upper bowel constraint (Table 4). PTV coverage ranged from 88.3% to 97.2% for these plans; original plan coverage was greater than 98%. For 4 of the 5 plans part of the seminal vesicles were identified as bowel, meaning the AI bowel was inside the 60 Gy PTV.

**Table 4.** Number of plans with clinician drawn OARs failing dose constraints, or plans with compromised PTV coverage when planned using AI OARs.

Number of plans	PTV and OAR Passing	Compromised PTV coverage	Clinician OAR exceeding dose constraints
20	15	5	1

**Figure 6.**
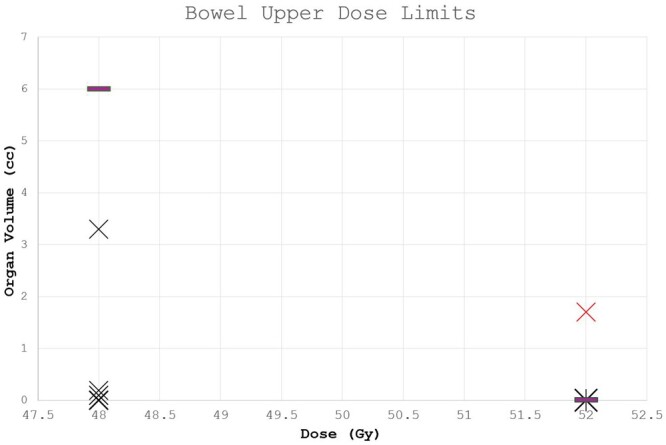
Volume of clinician drawn bowel receiving 48 and 52 Gy (ie, upper bowel dose constraints) for plans optimized with AI generated OARs. The purple line shows the maximum acceptable volume for each dose.

Dose to the clinically drawn rectum, bladder and femoral heads complied with local protocols for all plans, as did doses to bowel for all but the upper dose constraint on one plan. This failed with 1.7 cc of the bowel receiving 52 Gy (limit is 0.01 cc, Figure 6).

This study of the dosimetric data highlights that using unchecked contours could result in unnecessary compromising of the target coverage or incorrect reporting of dose to critical OARs.

## Discussion

### Timing

In this study we wanted to establish if Limbus, a software developed to generate contours for radiotherapy treatment planning, could offer efficiencies to prostate planning without introducing unacceptable risks to a patient’s treatment. The notable difference between this work and previous studies using Limbus is the inclusion of pelvic lymph nodes and bowel in the analysis.

The primary finding of this study was that Limbus reduced the time taken to contour the necessary structures for prostate plan by half. The biggest time saving was for outlining bowel: 7 minutes.

Zabel et al^13^ found that modifying Limbus bladder and rectum structures was 6.2 minutes quicker than drawing them *de novo*; similarly our experts saved 6.8 minutes. Wong et al^11^ compared expert timing for a prostate plan with the time Limbus took to generate its contours; they reported an average expert contouring time of 21 minutes. This is the same as our *de novo* prostate timings, giving validity to our timing data. Radici et al^19^ noted a combined time saving of 4 minutes when using Limbus to outline bladder, rectum and femoral heads: our time saving was double this. Doolan et al^5^ reported time savings of 34-42 minutes for contouring prostate and OARs for five different commercial AI software; these time savings were considerably larger than ours. However, it should be noted that only three patients were included in these two studies^5^^,^^19^, in comparison to the 30 used for this timing study. No other Limbus studies have compared pelvic node timings to date however Kiljunen et al^14^ performed this analysis using MVision, another commercial AI software. They reported a reduction in nodal contouring time from 20 to 8 minutes; compared to our reduction from 20 to 15 minutes. They reported a larger efficiency gain than us, however the time to outline nodal volumes *de novo* was comparable to ours, providing confidence in our timing data.

After comparing timing for 30 patients, it was evident that modifying contours was quicker than drawing them manually: therefore, we stopped the study. The contouring times reported here are comparable with those reported by other groups; furthermore, the time savings are similar to other studies (when direct comparisons can be made), giving validity to the data. It should be stated that timing data is very difficult to obtain in a busy radiotherapy clinic, presumably explaining why many studies focus on geometric similarity.

### Clinician scoring

Despite demonstrating that Limbus meaningfully shortened the contouring time required per prostate plan, it was interesting that the clinician satisfaction scoring of the AI contours was relatively low. All of the structures were classified as needing some editing, with femoral heads, bowel, and lymph nodes given scores of 1 (≥4 slices required editing). The scoring metric was selected as it is relatively objective, making it easier for the clinician to score. However, a potential weakness of this system is that a contour had to be classified as 1 if there was a difference in the superior or inferior border of four of more slices, which was routinely the case for the femoral heads and bowel. Clinicians, reported good in-slice agreement between themselves and Limbus for femoral heads, but graded them as 1 because Limbus contours went too inferior for our protocols. Modifications to the AI femoral heads were however very quick so the scoring potentially paints a more negative picture of “clinician satisfaction” than is the reality.

### Geometric similarity

We assessed the geometric similarity of the expert and auto- contours despite the short comings of these metrics^5^, as these are a commonly used tool to evaluate AI performance, are relatively simple to do and give some indication of similarity. However, our main analysis is not based on these results. Limbus and clinician drawn bladder structures showed near perfect agreement, which is comparable with DSC values reported for Limbus and other AI solutions^11^^,^^13^^,^^14^^,^^16^^,^^17^^,^^19^. Our DSC of 0.82 for the rectum is similar to other published data, with DSC scores varying between 0.78 and 0.87^11^^,^^13^^,^^14^^,^^16^^,^^17^^,^^19^. It is difficult to compare our DSC for prostate and seminal vesicle contours as the majority of published studies have separated seminal vesicles from prostate in their analysis; it did however have a relatively high DSC: 0.85.

The in-slice agreement between Limbus and the expert was good for all OARs, differences were seen in the levels used for each structure. Superior and inferior levels are likely to be clinic or country specific, depending which trial protocols or Atlases are followed meaning an AI software cannot be perfect for all clinics worldwide. European guidelines can differ from those in the USA, possibly causing some differences. However, Limbus are addressing this, sourcing modelling data from five continents for future releases.

These international variations and the individual patient anatomical variability should be considered when evaluating the reported performance of different AI solutions.

### Dosimetric study

The dosimetric study was intended to determine any potential risks of accidentally using unreviewed AI contours for treatment planning. It highlighted that target coverage could be unwittingly compromised and in rare cases OAR doses exceeded; emphasizing the importance of safe clinical implementation and careful expert review of AI structures. We have shown that there is a good initial agreement between Limbus and expert contours which gives clinicians a universal starting point for OAR contouring that may help to reduce inter-observer variability.

Based on these findings Limbus is now used routinely in our clinic. It acts as an aide to the clinicians, with all structures requiring review by a clinician before being approved. This practise is in line with recent recommendations published by the National Institute for Health and Care Excellence^23^.

## Conclusion

Although AI generated contours are imperfect, the wide variations in human anatomy and the known inter-observer variability^1^^,^^2^ imply that perfection may be difficult to model and a degree of realism is required, we should not let perfection get in the way of good.

Meaningful time savings are achieved when using Limbus AI contouring for prostate radiotherapy planning. The time savings of 13 minutes for prostate only and 26 minutes for prostate and nodal patients are proof of this.

In our clinic where we typically treat 200 prostate patients per year, Limbus frees up 60 hours of clinician time each year, enabling them to concentrate on other essential clinical tasks. The software has been successfully adopted into our planning pathway and is viewed as an extremely useful aide by our prostate doctors.

## References

[1] van der Veen J , Gulyban A, Willems S, Maes F, Nuyts S. Interobserver variability in organ at risk delineation in head and neck cancer. Radiat Oncol. 2021;16(1):120. 34183040 10.1186/s13014-020-01677-2 PMC8240214

[2] Berry SL , Boczkowski A, Ma R, Mechalakos J, Hunt M. Interobserver variability in radiation therapy plan output: Results of a single-institution study. Pract Radiat Oncol. 2016;6(6):442-449. 27374191 10.1016/j.prro.2016.04.005 PMC5099085

[3] Urago Y , Okamoto H, Kaneda T, et al Evaluation of auto-segmentation accuracy of cloud-based artificial intelligence and atlas-based models. Radiat Oncol. 2021;16(1):175. 34503533 10.1186/s13014-021-01896-1 PMC8427857

[4] Ginn JS , Gay HA, Hilliard J, et al A clinical and time savings evaluation of a deep learning automatic contouring algorithm. Med Dosim. 2023;48(1):55-60. 36550000 10.1016/j.meddos.2022.11.001

[5] Doolan PJ , Charalambous S, Roussakis Y, et al A clinical evaluation of the performance of five commercial artificial intelligence contouring systems for radiotherapy. Front Oncol. 2023;13. Accessed September 21, 2023. https://www.frontiersin.org/articles/10.3389/fonc.2023.1213068 10.3389/fonc.2023.1213068 PMC10436522 37601695

[6] Almberg SS , Lervåg C, Frengen J, et al Training, validation, and clinical implementation of a deep-learning segmentation model for radiotherapy of loco-regional breast cancer. Radiother Oncol. 2022;173:62-68. 35618100 10.1016/j.radonc.2022.05.018

[7] Shi F , Hu W, Wu J, et al Deep learning empowered volume delineation of whole-body organs-at-risk for accelerated radiotherapy. Nat Commun. 2022;13(1):6566. 36323677 10.1038/s41467-022-34257-x PMC9630370

[8] Taha AA , Hanbury A. Metrics for evaluating 3D medical image segmentation: analysis, selection, and tool. BMC Med Imaging. 2015;15(1):29. 26263899 10.1186/s12880-015-0068-x PMC4533825

[9] Chen W , Li Y, Dyer BA, et al Deep learning vs. atlas-based models for fast auto-segmentation of the masticatory muscles on head and neck CT images. Radiat Oncol. 2020;15(1):176. 32690103 10.1186/s13014-020-01617-0 PMC7372849

[10] Liu C , Gardner SJ, Wen N, et al Automatic segmentation of the prostate on CT images using deep neural networks (DNN). Int J Radiat Oncol Biol Phys. 2019;104(4):924-932. 30890447 10.1016/j.ijrobp.2019.03.017

[11] Wong J , Fong A, McVicar N, et al Comparing deep learning-based auto-segmentation of organs at risk and clinical target volumes to expert inter-observer variability in radiotherapy planning. Radiother Oncol. 2020;144:152-158. 31812930 10.1016/j.radonc.2019.10.019

[12] Cha E , Elguindi S, Onochie I, et al Clinical implementation of deep learning contour autosegmentation for prostate radiotherapy. Radiother Oncol. 2021;159:1-7. 33667591 10.1016/j.radonc.2021.02.040 PMC9444280

[13] Zabel WJ , Conway JL, Gladwish A, et al Clinical evaluation of deep learning and atlas-based auto-contouring of bladder and rectum for prostate radiation therapy. Pract Radiat Oncol. 2021;11(1):e80-e89. 32599279 10.1016/j.prro.2020.05.013

[14] Kiljunen T , Akram S, Niemelä J, et al A deep learning-based automated ct segmentation of prostate cancer anatomy for radiation therapy planning-a retrospective multicenter study. Diagnostics (Basel). 2020;10(11):959. 33212793 10.3390/diagnostics10110959 PMC7697786

[15] NDRS [Internet]. Radiotherapy Data Set (RTDS). Accessed September 22, 2023. https://digital.nhs.uk/ndrs/data/data-sets/rtds

[16] Savenije MHF , Maspero M, Sikkes GG, et al Clinical implementation of MRI-based organs-at-risk auto-segmentation with convolutional networks for prostate radiotherapy. Radiat Oncol. 2020;5(1):104. 10.1186/s13014-020-01528-0 PMC7216473 32393280

[17] Duan J , Bernard M, Downes L, et al Evaluating the clinical acceptability of deep learning contours of prostate and organs‐at‐risk in an automated prostate treatment planning process. Med Phys. 2022;49(4):2570-2581. 35147216 10.1002/mp.15525

[18] Wong J , Huang V, Wells D, et al Implementation of deep learning-based auto-segmentation for radiotherapy planning structures: a workflow study at two cancer centers. Radiat Oncol. 2021;16(1):101. 34103062 10.1186/s13014-021-01831-4 PMC8186196

[19] Radici L , Ferrario S, Borca VC, et al Implementation of a commercial deep learning-based auto segmentation software in radiotherapy: evaluation of effectiveness and impact on workflow. Life (Basel). 2022;12(12):2088. 36556455 10.3390/life12122088 PMC9782080

[20] The PACE trial: international randomised study of laparoscopic prostatectomy vs. stereotactic body radiotherapy (SBRT) and standard radiotherapy vs. SBRT for early stage organ-confined prostate cancer. *J Clin Oncol*. Accessed September 21, 2023. https://ascopubs.org/doi/abs/10.1200/JCO.2018.36.6_suppl.TPS153

[21] Taylor A , Rockall AG, Reznek RH, Powell MEB. Mapping pelvic lymph nodes: guidelines for delineation in intensity-modulated radiotherapy. Int J Radiat Oncol Biol Phys. 2005;63(5):1604-1612. 16198509 10.1016/j.ijrobp.2005.05.062

[22] Liu Z , Liu F, Chen W, et al Automatic segmentation of clinical target volume and organs-at-risk for breast conservative radiotherapy using a convolutional neural network. Cancer Manag Res. 2021;13:8209-8217. 34754241 10.2147/CMAR.S330249 PMC8572021

[23] 1 Recommendations. Artificial intelligence technologies to aid contouring for radiotherapy treatment planning: early value assessment. Guidance. NICE; 2023. Accessed September 29, 2023. https://www.nice.org.uk/guidance/hte11/chapter/1-Recommendations

